# Unidentified Neuronal Surface IgG Autoantibodies in a Case of Hashimoto's Encephalopathy

**DOI:** 10.3389/fimmu.2020.01358

**Published:** 2020-07-07

**Authors:** Marina Mané-Damas, Anita Vinke, Carolin Hoffmann, Shenghua Zong, Mario Losen, Peter C. Molenaar, Jan Damoiseaux, Suzanne Koudijs, Rob P. W. Rouhl, Pilar Martinez-Martinez

**Affiliations:** ^1^Department of Psychiatry and Neuropsychology, Maastricht University, Maastricht, Netherlands; ^2^School of Mental Health and Neuroscience, Faculty of Health, Medicine and Life Sciences, Maastricht University, Maastricht, Netherlands; ^3^Department of Neurology, Maastricht UMC+, Maastricht, Netherlands; ^4^Central Diagnostic Laboratory, Maastricht UMC+, Maastricht, Netherlands; ^5^Academic Center for Epileptology Kempenhaeghe/MUMC+, Heeze and Maastricht, Netherlands

**Keywords:** Hashimoto encephalopathy, autoimmune encephalitis, autoantibodies, case report, pathogenicity

## Abstract

Hashimoto's encephalopathy is an encephalitis of presumed autoimmune origin characterized by the presence of autoantibodies against thyroid proteins. We present a case of a young patient with pre-existing Hashimoto's thyroiditis and progressive cognitive complaints, absence-like episodes, and sporadic bilateral epileptiform frontal and frontotemporal activity. No abnormalities were observed during the neurological examination and on MRI. Antibodies to thyroid peroxidase (TPO) were elevated and remained positive while the symptoms were present. Levothyroxine and methylprednisolone did not ameliorate the complaints. Subsequent treatment with high-dose intravenous immunoglobulins (IVIG) led to improved cognitive functions and to the disappearance of the absence-like-episodes. Patient's serum, but not CSF, gave a characteristic IgG-specific hippocampal pattern in rat brain immunohistochemistry; this immunoreactivity was maintained after specific and complete depletion of TPO antibodies. Serum IgG bound to primary neurons in cell culture, likely targeting a yet unidentified neuronal surface antigen. The clinical response to IVIG suggests but does not prove, that the circulating novel autoantibodies may induce the encephalopathy. It would be of interest to investigate more patients with Hashimoto's encephalopathy for the presence of neuronal surface autoantibodies, to define their role in the disease and their target antigen(s).

## Background

Autoimmune encephalitides are debilitating disorders characterized by a rapid progression of prominent neuropsychiatric manifestations, associated with autoantibodies against neuronal cell-surface proteins, ion channels, or neurotransmitter receptors, and a good response to immunotherapy (1).

Hashimoto's encephalopathy, also known as steroid-responsive encephalopathy associated with autoimmune thyroiditis (SREAT), is a rare disorder characterized by a variable presentation of neurological and psychiatric manifestations, the presence of anti-thyroid antibodies and by a clinical response to steroids (1). However, as thyroid antigens are mainly expressed in the thyroid, this would not explain the presence of cognitive decline and neurological manifestations (2). Recently, pathogenic autoantibodies to neuronal receptors have been identified, co-occurring in some cases with glutamic acid decarboxylase 65 (GAD65) or thyroid peroxidase (TPO) antibodies. The co-occurrence of both autoantibodies may result in misdiagnosis of the patient.

Here, we describe a young patient with suspected autoimmune encephalitis presenting with unidentified neuronal surface autoantibodies and concomitant TPO antibodies, who modestly responded to immunosuppressive treatment.

## Case Presentation

**Figure 1:**
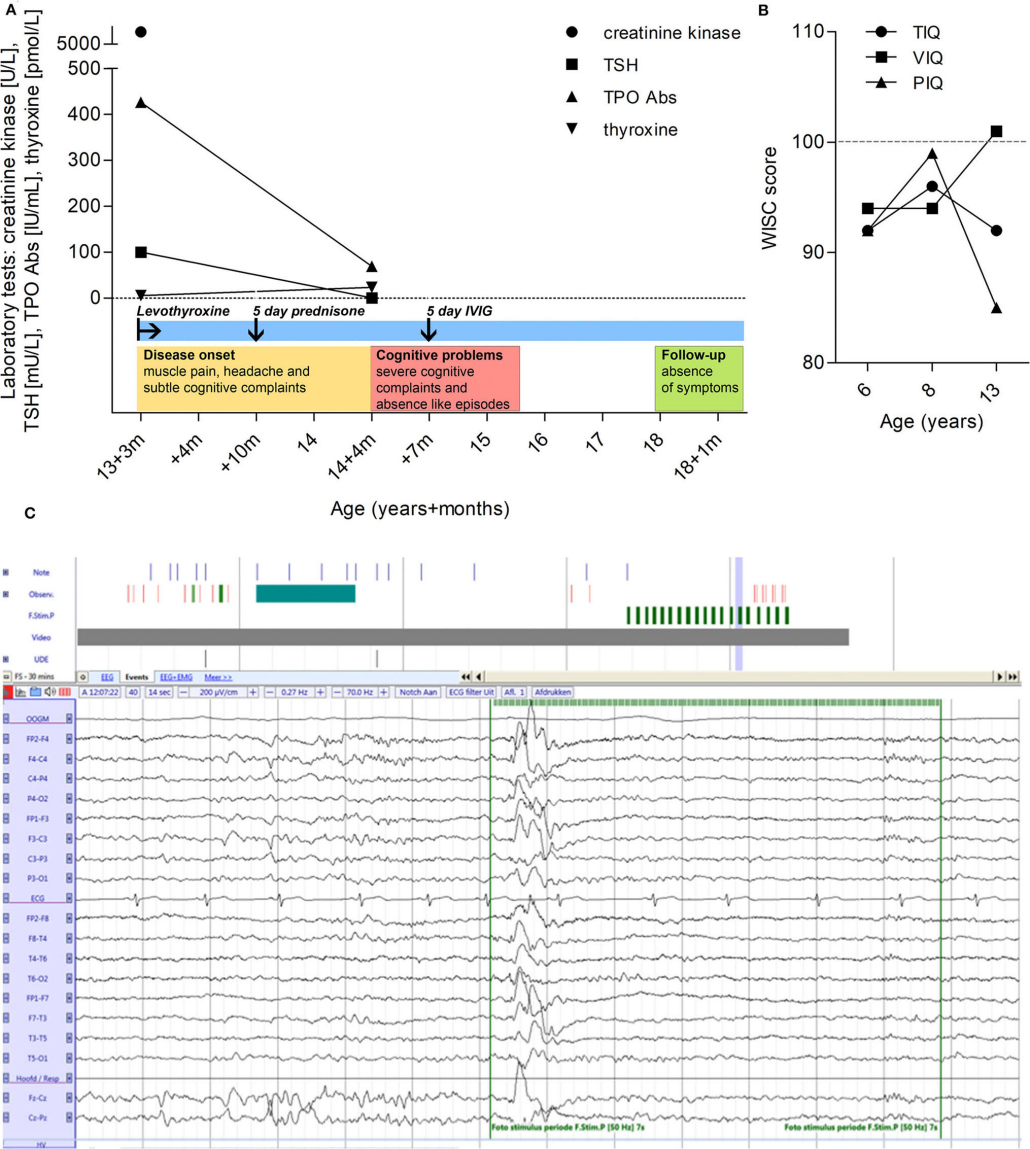
Clinical timeline, IQ, and EEG. **(A)** Clinical timeline representing the evolution of serological values of creatinine kinase, thyroid-stimulating hormone (TSH), TPO autoantibodies and thyroxine levels. Treatment intervention is also illustrated as well as qualitative disease progression. **(B)** IQ progression by Wechsler intelligence scale for children. Total IQ (TIQ), verbal IQ (VIQ), and performance IQ (PIQ) at the age of 6, 8, and 13. The average score for the test is 100, and any score between 90 and 109 is considered to be in the average intelligence range. **(C)** EEG showing generalized spike and wave discharge with right frontal dominance during photic stimulation with 50 Hz.

A 13-year-old boy with a previous history of Hashimoto's thyroiditis presented with muscle pain, dry skin, and subtle memory complaints. A scheme of the clinical events of this case report is shown in Figure 1A. Serological analysis at that time, revealed high levels of creatinine kinase (5,105 U/L; normal value <171 U/L), thyroid-stimulating hormone (TSH) (>100 mU/L; normal value 0.50–3.40 mU/L), anti-TPO (426.2 IU/mL; normal value <100 IU/mL), whereas thyroxine levels were low (<5.2 pmol/L; normal value 11.5–17.7 pmol/L). Treatment was started with levothyroxine 100 mcg, once daily, after which his thyroxine levels normalized and muscle pain and skin dryness improved. However, 6 months after the diagnosis, the cognitive complaints worsened and the patient received a 5-day course of methylprednisolone, which did not alter his symptoms. Next, his school performance declined in quality, indicating a further worsening in his cognitive abilities. A neuropsychological test showed no remarkable differences beside a subtle decline in his performance intelligence quotient (IQ) (Figure 1B). One year after his first memory complaints started, the patient was referred to our academic hospital for further investigation with the suspicion of an autoimmune encephalitis.

Family history was positive for hypothyroidism and high TPO autoantibody levels on the mother's side. Neurological examination showed no focal deficits or other abnormalities, and brain MRI was unremarkable. At time of admission to our pediatric neurology department, the patient was suffering from amnesia and had long lapses of concentration. Generalized absence seizures were suspected. However, repeated EEG tests, including a 24-h registration, only revealed sporadic bilateral frontal and frontotemporal activity with some epileptiform features, without clinical correlation (Figure 1C). Therefore, the absence-like episodes were not considered to be of epileptic origin. Repeated serological laboratory tests showed normal levels of TSH (0.5 mU/L), presence of TPO antibodies (69 IU/mL), and moderately elevated thyroxine levels (23.4 pmol/L). The lack of response to corticosteroids made a Hashimoto's encephalopathy (SREAT) less likely. Because of the ongoing subjective cognitive decline an alternative cause of this encephalopathy was considered. Further investigation revealed normal cerebrospinal fluid (CSF) cell count, glucose, and protein levels, and autoantibodies known to cause autoimmune encephalitis including N-methyl-D-aspartate receptor (NMDAR), α-amino-3 hydroxy-5-methyl-4-isoxazolepropionic acid receptor (AMPAR), γ-aminobutyric acid receptor subunits B (GABA_B_R), leucine-rich glioma-inactivated 1 (LGI1), and contactin-associated protein-like 2 (CASPR2), were undetectable in serum and CSF (autoimmune encephalitis Mosaic 1, Euroimmun). Myelin oligodendrocyte glycoprotein (MOG) autoantibodies were negative in serum (Cell Based FACS assay, Sanquin, Amsterdam). The clinical symptoms in combination with these results, led to a working diagnosis of a possible (seronegative) autoimmune encephalopathy. Therefore, the patient was treated with a 5-day course of high-dose intravenous immunoglobulins (IVIG). After the treatment, the cognitive decline stabilized and the absence-like episodes ceased. He finished secondary school (HAVO) and no further deterioration has been observed since the initial manifestations, 5 years ago.

## Antibody and Antigen Characterization

**Figure 2:**
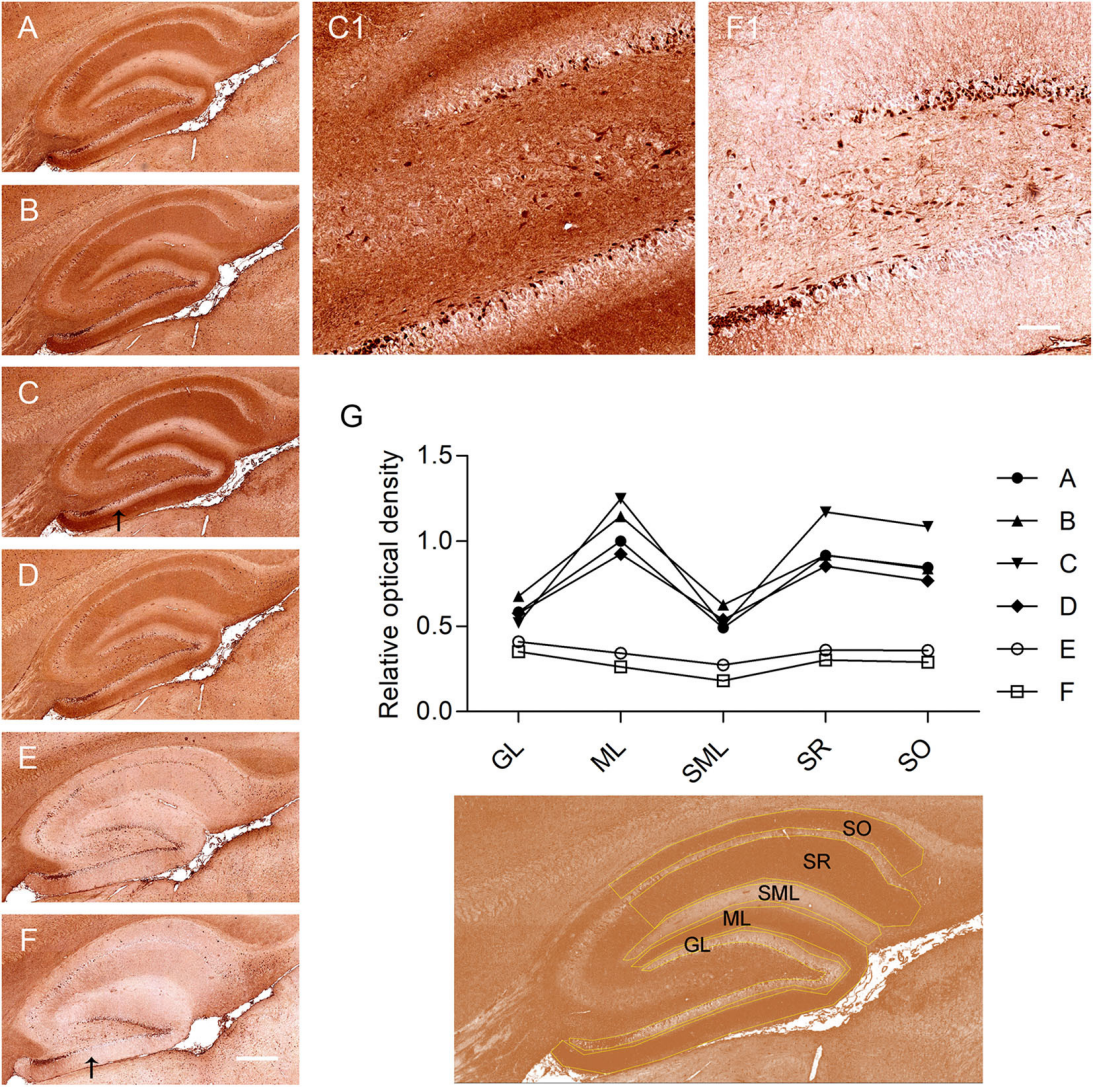
Identification and quantification of a novel neuropil-like hippocampal pattern in rat brain sections. **(A–F)** Rat brain immunohistochemistry IgG reactivity of serum. **(A–D)** Brain immunoreactivity of sera of the Hashimoto's encephalopathy patient case at different time points: **(A)** At first presentation, **(B)** 1 month after first presentation, **(C)** 1 year after first presentation, and **(D)** after IVIG infusion. **(E)** brain immunoreactivity of serum from a patient with epilepsy and TPO autoantibodies. **(F)** brain immunoreactivity of serum from a non-disease control. The arrows in panels **(C,F)** point at areas shown at higher magnification in panel **(C1,F1)**, respectively. Note the predominance of a neuropil-like pattern in panel **(C1)**. **(G)** Relative optical density quantification in different areas of the hippocampus, outlined in yellow. SO, stratum oriens; SR, stratum radiatum; SML, stratum lacunosum; ML, molecular layer of dentate gyrus; GL, granule cell layer. Scale bars are 100 and 20 μm.

**Figure 3:**
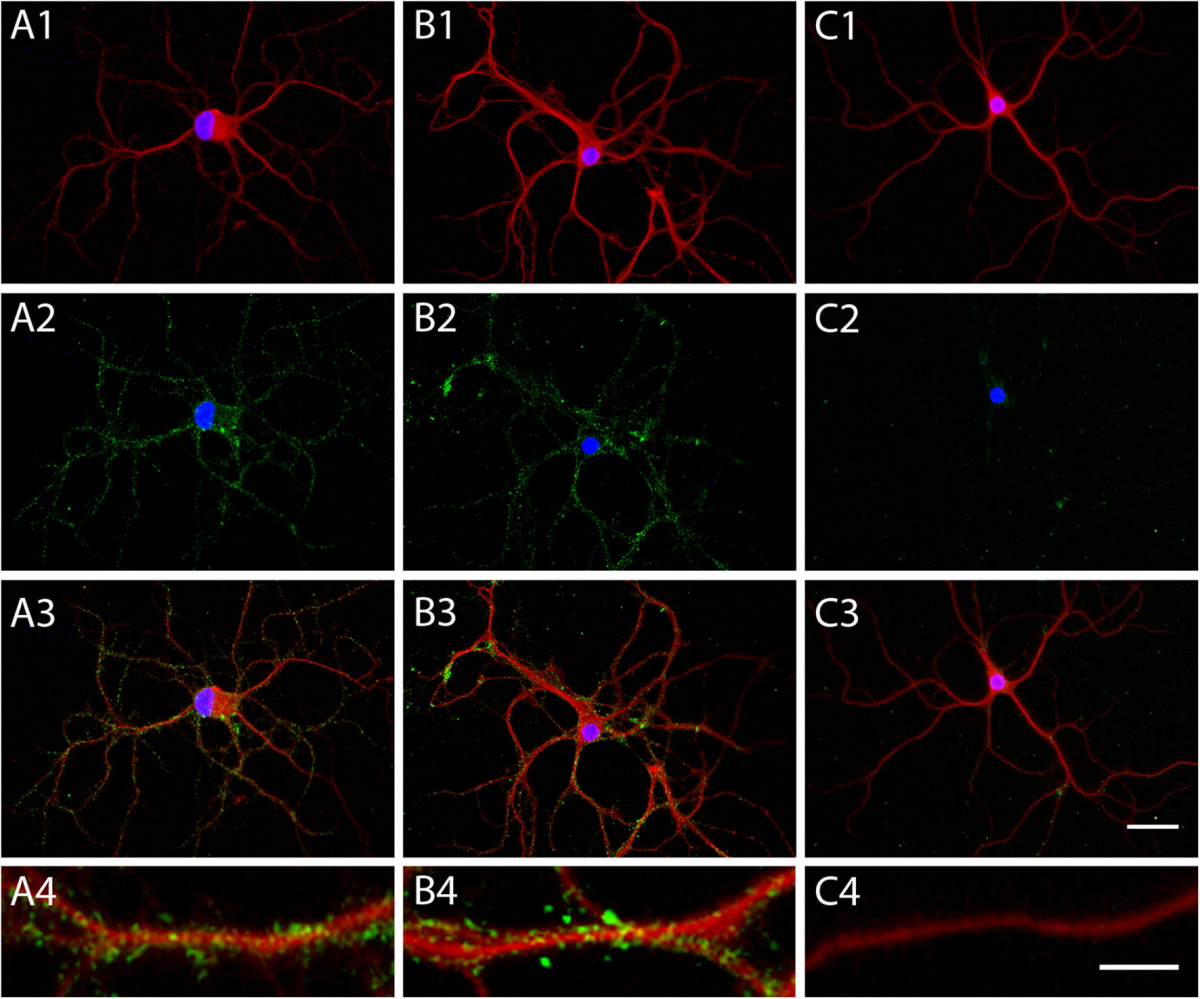
Patient's autoantibodies recognize an unidentified neuronal surface antigen in cultured primary hippocampal neurons. Immunofluorescent photomicrographs of cultured, live primary hippocampal neurons. Human IgGs are stained in green fluorescence, microtubules (MAP2) in red fluorescence, and cell nuclei (Hoechst DNA stain) in blue fluorescence. Serum immunostaining of **(A)** a patient with DPPX antibodies, as a control, **(B)** the Hashimoto's encephalopathy patient case, and **(C)** a non-disease control. **(A1,B1,C1)** show the merge of nuclear and dendrite staining, **(A2,B2,C2)** show the merge of nuclear and human IgG staining, **(A3,B3,C3)** show the merge of all three fluorescent stainings. **(A4,B4,C4)** show the immunofluorescence staining of a dendrite at higher magnification. Scale bars are 50 μm.

Rat brain sections and primary cell cultures from rat embryo hippocampus were used to detect IgG autoantibodies to neuronal antigens (3). A strong immunoreactivity of serum antibodies to the middle region of the dentate gyrus and the molecular layer of the cerebellum was identified, with a titer of 1:1,600. Pyramidal neurons and GABA-ergic interneurons in the hippocampus were labeled and the hippocampal neuropil-like pattern was present using sera corresponding to different disease time points (Figures 2A–D). In contrast, rat brain immunoreactivity of sera from a TPO-epilepsy control serum, and a non-disease control, was low (Figures 2E,F). To quantify the intensity of the rat brain stainings, we selected different areas of the hippocampus including the stratum oriens, the stratum radiatum, the stratum lacunosum-moleculare, the molecular layer of dentate gyrus, and the granule cell layer. The optical density of the patient's serum immunoreactivity at all investigated time points was consistently high in these hippocampus regions, specially in the molecular layer of the dentage gyrus, the stratum radiatum, and the stratum oriens (Figure 2G). Interestingly, this immunoreactivity was highest at the time point corresponding to one year after onset, when the patient was admitted to our neurology department. Autoantibody reactivity to a surface antigen was confirmed using live rat primary hippocampal neurons (Figure 3). However, live and fixed cell-based assays for NMDA, AMPA, GABA_A_, GABA_B_ receptors as well as LGI1, and CASPR2 membrane proteins and the intracellular proteins GAD65 and GAD67 were negative. The staining using CSF was negative in cultured live rat hippocampal neurons, in the rat immunohistochemistry, and in the abovementioned cell-based assays.

**Figure 4:**
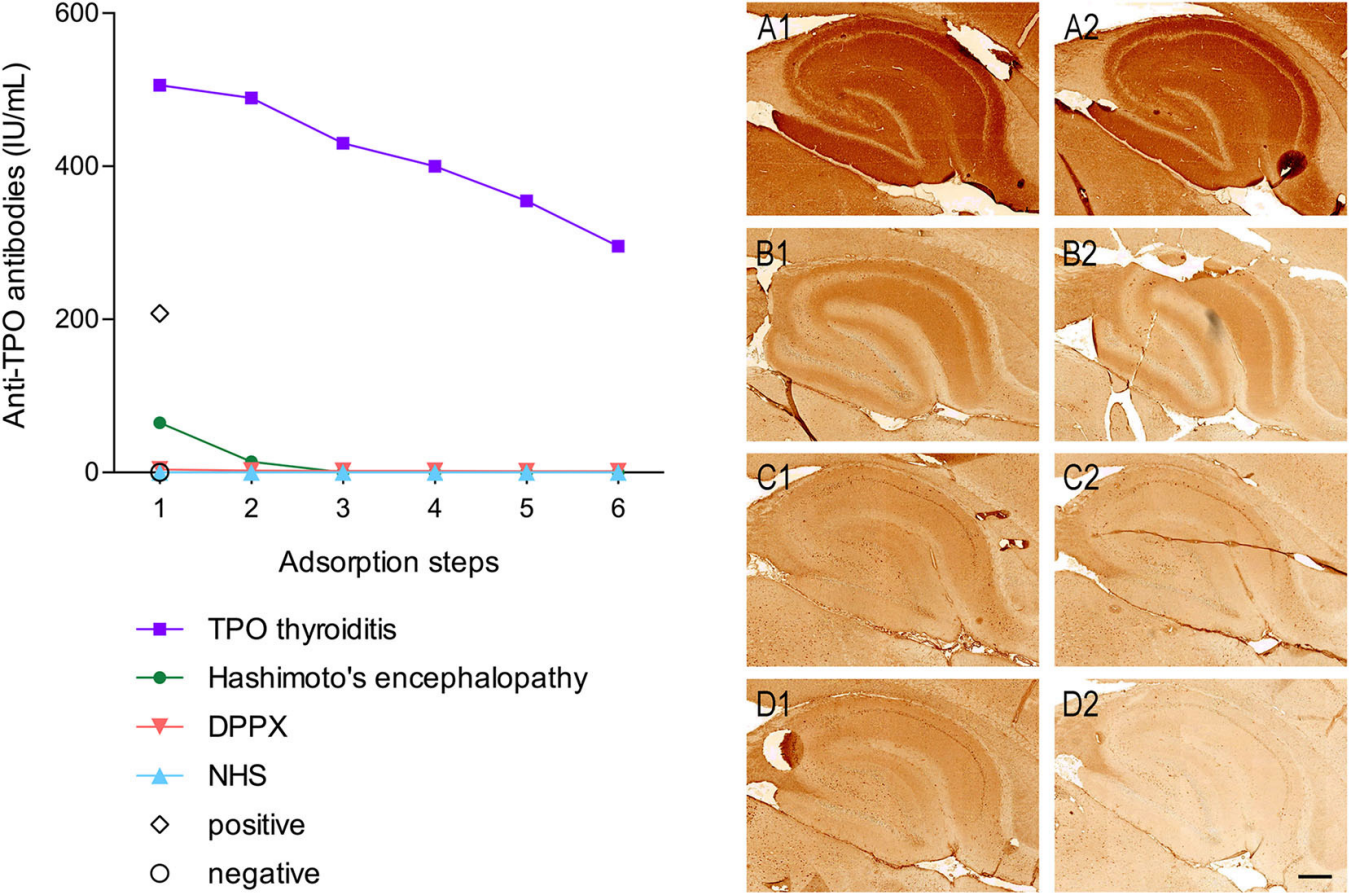
TPO autoantibodies are not responsible for the neuronal reactivity and TPO immuno-adsorption shows co-existence of autoantibodies. Graph on the left panel: TPO antibody levels (IU/mL) at six consecutive steps of immune-adsorption using wells coated with recombinant human TPO protein. TPO antibody was depleted from the sera of the following patients: a patient with TPO antibody-positive thyroiditis (purple squares), the Hashimoto's encephalopathy patient (green closed circles), a patient with autoimmune encephalitis and dipeptidyl-peptidase-like protein 6 (DPPX) antibodies (magenta inverted triangles), and a non- disease human serum control (NHS) (blue triangles). A positive (open diamond) and a negative (open circle) calibrator control were included without absorption. Right panel: Rat brain immunohistochemistry using non-immuno-adsorbed sera **(A1–D1)** and immune-adsorbed sera **(A2–D2)** from panel **(A)** a patient with DPPX antibodies, **(B)** the Hashimoto's encephalopathy patient, **(C)** a patient with TPO positive antibody thyroiditis, and **(D)** a non-disease control. Scale bar is 100 μm.

The coexistence of additional autoantibodies in the serum besides anti-TPO was confirmed after six consecutive immune-adsorption steps, using TPO-coated microtiter wells from the anti-TPO ELISA (IgG) kit (Euroimmun; EA 1012-0961 G). Anti-TPO IgG levels were reduced from 87.2 to 0 IU/mL. However, the TPO antibody depleted serum retained the same brain IgG immunoreactivity pattern (Figure 4).

## Discussion

The presence of serum autoantibodies against thyroid proteins, including TPO, is one of the key diagnostic criteria for Hashimoto's encephalopathy (1). Clinically, patients can present with a broad range of symptoms, including seizures, myoclonus, stroke-like episodes with focal neurological, and psychiatric manifestations such as hallucinations, impaired cognition, or even dementia. In this particular case, a subjective cognitive decline and absence-like episodes occurred, which persisted after adequate supplementation of thyroid hormone and treatment with methylprednisolone.

Hashimoto's encephalopathy is known as steroid-responsive encephalopathy associated with autoimmune thyroiditis (SREAT) due to the high rate of response to steroids (1). Even though more than 95% of patients show a good response to steroids (4), some patients require additional immunosuppressive therapies including IVIG, cyclosporine, cyclophosphamide, and rituximab (5).

TPO is a membrane-bound glycoprotein, mainly expressed in the thyroid. Thyroid autoantibodies are present in more than 10% of the healthy population, as well as in patients with autoimmune encephalopathies, questioning their causative role in the pathology (6). A study showed that ~16% of autoimmune encephalitis patients with GABA_A_R antibodies have coexisting TPO autoantibodies (5). Furthermore, cognitive impairment and other neuropsychiatric manifestations in these patients did not correlate with the presence of TPO antibodies as the fluctuations of the autoantibody levels did not correlate with the clinical manifestations (7).

The presence of TPO autoantibodies in CSF is not included in the diagnostic criteria for Hashimoto's encephalopathy. Hence, CSF of our patient was not initially tested for autoantibodies against thyroid antigens. Only a few studies have analyzed thyroid autoantibodies and circulating immune complexes in the CSF, which were reported in a small cohort of Hashimoto's encephalopathy patients but not in a control group (8). Furthermore, we did not find nor identify known autoantibodies associated with autoimmune encephalitis in the CSF; moreover, no reactivity of CSF against neuronal antigens was detected by rat brain immunohistochemistry. Undetectable autoantibody levels in the CSF of patients with neuro-psychiatric manifestations could result from an immune adsorption effect within the brain where the autoantigen is expressed (9). Other factors that could contribute are low autoantibody levels crossing the blood brain barrier, in combination with the use of techniques with high detection limit. Nevertheless, low autoantibody levels in the brain could have an effect when they have high affinity and a wide range of effector functions.

Co-occurrence of TPO autoantibodies with AMPAR, NMDAR, GABA_A_R, GABA_B_R, LGI1, and MOG autoantibodies has been described in patients with very diverse clinical phenotypes, with a predominance of epilepsy (5, 10–12). These findings are supported by this patient's case report, where the TPO autoantibodies were not responsible for the brain-specific IgG (immuno)reactivity, implying the presence of other autoantibodies targeting a neuronal antigen. Lack of reactivity against the currently known neuronal antigens targeted in autoimmune brain disorders and the lack of similarity in the known hippocampal patterns preclude at present the identification of the pathogenic target (1).

Tuzun et al. (10) described the presence of autoantibodies against novel targets in two out of eight patients with limbic encephalitis and co-occurring thyroid autoantibodies. Presence of IgG reactivity was also identified in Hashimoto's encephalopathy patient serum using human and mouse brain sections while the results from patients with Hashimoto's thyroiditis were negative (13). Various studies have described the presence of unidentified autoantibodies against neuronal (surface) antigens with a potential pathogenic role in other pathologies (14) (S. Zong, unpublished results).

The clinical manifestations displayed by the patient described in this case report and the conspicuous hippocampal immunoreactivity pattern of the patient's sera, are novel to the best of our knowledge.

All-in all, TPO autoantibodies in our patient seem to be mere bystanders rather than having a causative, pathogenic role in Hashimoto's encephalopathy. The diagnosis of this encephalopathy remains controversial, since some patients do not meet all diagnostic criteria nor respond to steroids. It would be desirable to study the presence of neuronal surface autoantibodies in both serum and CSF in additional cases of suspected Hashimoto's encephalopathy in order to analyze their putative contribution to the pathology. A better understanding of the pathogenic mechanisms and co-occurrence of autoantibodies will contribute to aiming therapy at the right targets.

## Ethics Statement

The Hashimoto's encephalopathy patient's samples, data and permit for the publication of this case report were collected with written informed consent by the parents, according to the national and institutional ethical guidelines, in accordance with the Declaration of Helsinki. Additionally, the use of other human material reported within this case report was approved by the medical ethical committee of the Maastricht University Medical Center+.

## Author Contributions

AV, SK, and RR provided clinical information. JD provided the samples. MM-D, CH, SZ, PCM, ML, and PM-M designed the experiments and interpreted the data. MM-D performed the research. MM-D, AV, and PM-M drafted the manuscript. All authors revised the manuscript and gave critical feedback.

## Conflict of Interest

The authors declare that the research was conducted in the absence of any commercial or financial relationships that could be construed as a potential conflict of interest.

## References

[1] Graus F Titulaer MJ Balu R Benseler S Bien CG Cellucci T. A clinical approach to diagnosis of autoimmune encephalitis. Lancet Neurol. (2016) 15:391–404. 10.1016/S1474-4422(15)00401-9 26906964 PMC5066574

[2] Laurent C Capron J Quillerou B Thomas G Alamowitch S Fain O. Steroid-responsive encephalopathy associated with autoimmune thyroiditis (SREAT): Characteristics, treatment and outcome in 251 cases from the literature. Autoimmun Rev. (2016) 15:1129–33. 10.1016/j.autrev.2016.09.008 27639840

[3] Hoffmann C Zong S Mané-Damas M Molenaar PC Losen M Titulaer MJ. Absence of autoantibodies against neuronal surface antigens in sera of patients with psychotic disorders. JAMA Psychiatry. (2019) 77:322–5. 10.1001/jamapsychiatry.2019.3679 31746971 PMC6902152

[4] Chong JY Rowland LP Utiger RD Hashimoto encephalopathy: syndrome or myth? JAMA Neurol. (2003) 60:164–71. 10.1001/archneur.60.2.164 12580699

[5] Petit-Pedrol M Armangue T Peng X Bataller L Cellucci T Davis R. Encephalitis with refractory seizures, status epilepticus, and antibodies to the GABAA receptor: a case series, characterisation of the antigen, and analysis of the effects of antibodies. Lancet Neurol. (2014) 13:276–86. 10.1016/S1474-4422(13)70299-0 24462240 PMC4838043

[6] Hollowell JG Staehling NW Flanders WD Hannon WH Gunter EW Spencer CA. Serum TSH, T4, and thyroid antibodies in the United States population (1988 to 1994): National Health and Nutrition Examination Survey (NHANES III). J Clin Endocrinol Metab. (2002) 87:489–99. 10.1210/jcem.87.2.8182 11836274

[7] Napthali K Boyle M Tran H Schofield PW Peel R McEvoy M. Thyroid antibodies, autoimmunity and cognitive decline: is there a population-based link? Dement Geriatr Cogn Dis Extra. (2014) 4:140–6. 10.1159/000362716 24987403 PMC4067731

[8] Ferracci F Moretto G Candeago RM Cimini N Conte F Gentile M. Antithyroid antibodies in the CSF. Their role in the pathogenesis of Hashimoto's encephalopathy. Neurology. (2003) 60:712–4. 10.1212/01.WNL.0000048660.71390.C6 12601119

[9] Castillo-Gomez E Kästner A Steiner J Schneider A Hettling B Poggi G. The brain as immunoprecipitator of serum autoantibodies against N-Methyl-D-aspartate receptor subunit NR1. Ann Neurol. (2016) 79:144–51. 10.1002/ana.24545 26505629

[10] Tuzun EEE Durmus H Brenner T Turkoglu R Kurtuncu M Lang B Autoantibodies to neuronal surface antigens in thyroid antibody-positive and -negative limbic encephalitis. Neurol India. (2011) 59:47–50. 10.4103/0028-3886.76857 21339658

[11] Chen K-A Brilot F Dale RC Lafferty AR Andrews PI. Hashimoto's encephalopathy and anti-MOG antibody encephalitis: 50 years after Lord Brain's description. Eur J Paediatr Neurol. (2017) 21:898–901. 10.1016/j.ejpn.2017.06.002 28694134

[12] Armangue T Olivé-Cirera G Martínez-Hernandez E Sepulveda M Ruiz-Garcia R Muñoz-Batista M. Associations of paediatric demyelinating and encephalitic syndromes with myelin oligodendrocyte glycoprotein antibodies: a multicentre observational study. Lancet Neurol. (2020) 19:234–46. 10.1016/S1474-4422(19)30488-0 32057303

[13] Oide T Tokuda T Yazaki M Watarai M Mitsuhashi S Kaneko K. Anti-neuronal autoantibody in Hashimoto's encephalopathy: neuropathological, immunohistochemical, and biochemical analysis of two patients. J Neurol Sci. (2004) 217:7–12. 10.1016/j.jns.2003.08.005 14675602

[14] Mané-Damas M Hoffmann C Zong S Tan A Molenaar PC Losen M. Autoimmunity in psychotic disorders. Where we stand, challenges and opportunities. Autoimmun Rev. (2019) 18:102348. 10.1016/j.autrev.2019.102348 31323365

